# Emergence of a Novel Dengue Virus Serotype-2 Genotype IV Lineage III Strain and Displacement of Dengue Virus Serotype-1 in Central India (2019–2023)

**DOI:** 10.3390/v17020144

**Published:** 2025-01-23

**Authors:** Ashish Kumar Yadav, Rashmi Chowdhary, Arshi Siddiqui, Anvita Gupta Malhotra, Jagat R. Kanwar, Ashok Kumar, Debasis Biswas, Sagar Khadanga, Rajnish Joshi, Abhijit Pakhare, Sudhir Kumar Goel

**Affiliations:** 1 Department of Biochemistry, All India Institute of Medical Sciences, Bhopal 462 026, Madhya Pradesh, India; ashish.phd2019@aiimsbhopal.edu.in (A.K.Y.); jagat.biochemistry@aiimsbhopal.edu.in (J.R.K.);; 2 Department of Biotechnology, Barkatullah University, Bhopal 462 026, Madhya Pradesh, India; 3 Department of Microbiology, All India Institute of Medical Sciences, Bhopal 462 026, Madhya Pradesh, India; anvitagupta16@gmail.com (A.G.M.);; 4 Department of Medicine, All India Institute of Medical Sciences, Bhopal 462 026, Madhya Pradesh, India; 5 Community and Family Medicine, All India Institute of Medical Sciences, Bhopal 462 026, Madhya Pradesh, India

**Keywords:** dengue, DENV, mosquitoes, Bayesian analysis, outbreak prediction

## Abstract

Dengue fever remains a significant public health concern in tropical regions, including Central India, where outbreaks are frequent and associated with high morbidity and mortality. This study investigated the dynamics of dengue virus transmission and evolution in Central India from 2019 to 2023, focusing on the emergence of new strains and their impact on outbreak patterns. For this, 40 mosquito pools and 300 patient samples were recruited for the study. Phylogenetic and Bayesian evolutionary analyses performed on CPrM region and whole genome sequences generated by Sanger and Illumina sequencing, respectively, revealed the emergence and predominance of a novel DENV-2 genotype IV lineage III strain in the 2019 and 2023 outbreaks, which displaced the previously circulating DENV-1 genotype responsible for the 2016–2017 outbreak. Despite pre-existing DENV-1 neutralizing antibodies in the community (67 healthy volunteers), the novel DENV-2 strain exhibited higher viral loads and a greater reproduction number (R0), contributing to rapid disease spread. Molecular clock and Shannon entropy analyses suggest that DENV evolution occurred within the mosquito vector, driven by natural selection. Our findings highlight the importance of continuous DENV surveillance, including genetic characterization in both vectors and hosts, to understand viral evolution and predict future outbreaks. Rapid urbanization and inadequate sanitation in densely populated regions like India create ideal breeding grounds for mosquitoes, facilitating the introduction and establishment of novel DENV strains. Interrupting the vector–DENV–host cycle through targeted interventions is crucial for effective dengue control.

## 1. Introduction

Dengue is the most prevalent acute febrile illness transmitted by arthropods, caused by the dengue virus, with approximately 390 million new cases reported annually [1]. Female *Aedes* mosquitoes carrying the dengue virus (DENV) are the main vector of transmission [2]. There are four different serotypes of DENV: DENV-1, DENV-2, DENV-3, and DENV-4. Based on genetic and antigenic differences, these serotypes are further divided into different genotypes [3,4] with a ~30% inter-serotype variability [5,6]. Clinical signs and symptoms of DENV infections range from low-grade fever to severe hemorrhagic fever and even death [7]. The symptoms usually appear 5–8 days after an incubation period of 3–15 days. Clinical manifestations of dengue can range from an infection with no symptoms to a serious, potentially fatal illness if left untreated [8]. Expanded dengue syndrome, dengue fever, dengue hemorrhagic fever (DHF), dengue shock syndrome (DSS), undifferentiated febrile disease, and atypical dengue are the classifications used to describe cases [9]. Children, expectant mothers, and the elderly, particularly those with concomitant conditions like diabetes or hypertension, are among the groups most at risk of contracting dengue.

Diverse climatic conditions make the Indian subcontinent an epicenter for dengue disease, with 33 million infections [1], while in Central India, dengue epidemics have been occurring since 2009 [10,11,12]. Although previous studies have highlighted the importance of distinct serotypes and genotypes in determining the likelihood of an epidemic, little is known about the underlying genotype and serotype of the dengue outbreak [13].

Antibody-dependent enhancement (ADE) remains underexplored in this endemic region. Nonetheless, studies have demonstrated that subsequent infections with distinct DENV serotypes raise the chance of developing severe disease by three to four times, and various DENV serotypes co-circulate in endemic locations [14]. Higher plasma viremia has been linked to severe dengue sickness [15]. However, some studies showed that viremia is not the main driver of inflammation after a dengue infection, as it was higher in individuals with a primary infection [16].

There is still some uncertainty surrounding how new dengue virus strains emerge and how viremia levels in both humans and mosquito vectors influence disease severity. To better understand the frequency and impact of emerging viral shifts, thorough research is essential. Until an effective vaccine becomes widely available, controlling mosquito populations remains the primary method for reducing dengue transmission. Variations in the dengue virus’s genetic makeup contribute to differences in virulence and transmission between serotypes, leading to diverse patterns of disease severity. Consequently, monitoring the molecular characteristics of circulating DENV genotypes and serotypes is vital for predicting and managing outbreaks. Additionally, studying the molecular epidemiology of DENV in both human and mosquito populations is critical for improving epidemic forecasting and guiding public health interventions. 

From September 2019 to 2023, this study examined the seroprevalence and genetic epidemiology of DENV in both hosts and vectors in Bhopal, a dengue-endemic region in Central India. This research aimed to bridge the knowledge gap regarding how high viremia, driven by a high reproductive number (R0) in hosts/vectors, and neutralizing antibody responses may predict future dengue outbreaks in endemic areas. Molecular characterization revealed the emergence of new genotypes and serotypes in vectors, influencing strain competition and transmission dynamics. This contributes to the cyclical outbreaks observed every two to three years, enhancing our understanding of improved outbreak management.

## 2. Materials and Methods

### 2.1. Study Area, Study Period, and Sample Sources

**Figure 1:**
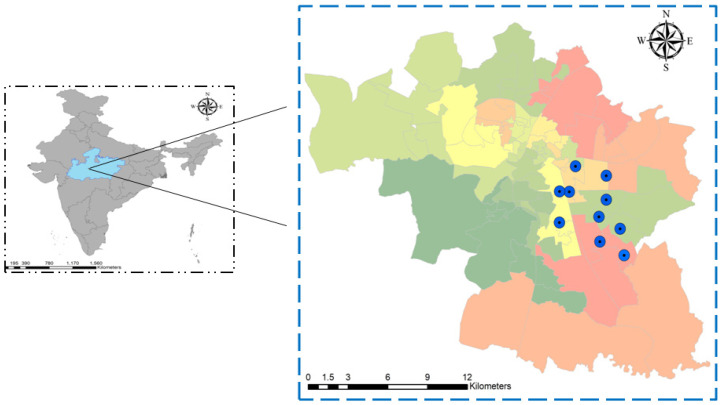
Map of Bhopal City showing hot-spot wards (52–61) for dengue cases and for mosquito sample collection (Blue dot represents the sample collection wards).

This study complied with the Declaration of Helsinki and was approved by the Institutional Human Ethics Committee (IHEC Ref No-2019/PhD/Jan/19/04) at AIIMS, Bhopal, India. After obtaining written informed consent, 3 mL of blood from 300 patients showing suspected dengue symptoms was collected in a serum separator tube (SST/yellow vial) and in an EDTA vial from the OPD (Out Patient Department). The samples were transported to the laboratory for centrifugation to separate the serum and stored at −80 °C for further analysis. Of the 300 samples, serum was successfully isolated from 280 samples, and 92 of them tested positive for the DENV NS1 antigen. Two samples were disqualified because they were co-infected with both DENV and Chikungunya virus. The city of Bhopal in Madhya Pradesh, Central India, with a population of 2,553,000, was chosen for the current study. Gulmohar Colony, Bagsewaniya, Arera Colony, Baghmugaliy, Barkheda, Saket Nagar/Shakti Nagar, Kasturba Nagar, Bharat Heavy Electrical Limited (BHEL), and Maharana Pratap Nagar represents ward number 52 to 61 (Figure 1) and were identified as hot-spots for this study due to the high dengue case density in these areas. A total of 15% (0.37 million) of the city’s population lives in these locations, which were chosen as catchment areas to gather samples from dengue patients, vector samples, and samples for neutralizing antibodies testing.

### 2.2. Patients Clinical Details

After considering the inclusion and exclusion criteria, 300 patients with warning signs of a headache, myalgia, fever, arthralgia, rash, vomiting/nausea, retro-orbital pain, hematocrit (%), and thrombocytopenia were recruited in the study. A Complete Blood Count (CBC) and a biochemical parameter (LFT) were also performed for the patients (Supplementary Table S1).

### 2.3. Serological Diagnosis of Dengue Virus

**Figure 2:**
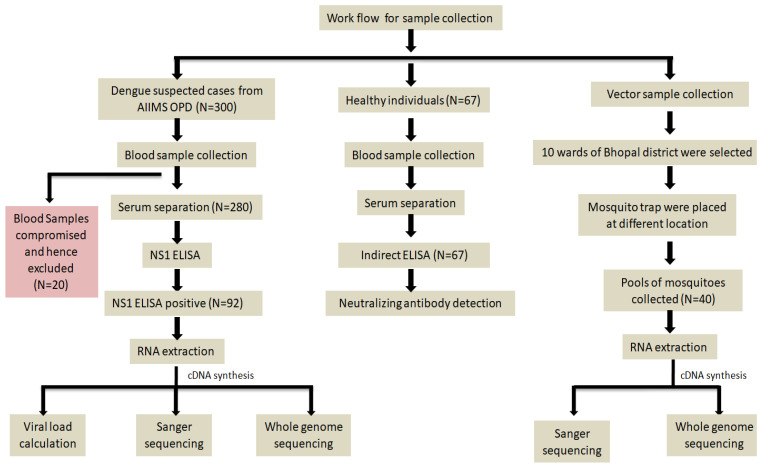
Flow chart of the sample recruitment.

An ELISA was performed to detect the presence of dengue NS1 protein in 280 serum samples by using a commercially available kit, Dengue NS1 Ag Microlisa ELISA kit (J. Mitra & Co. Pvt. Ltd., New Delhi, India; Ref. No. IR031096), following the manufacturer’s protocol (Figure 2).

### 2.4. Mosquito Sample Collection and Processing

Mechanical aspirators and the Biogents BG-Sentinel-2-Mosquito-Trap (Biogents, Regensburg, Germany) were used to collect mosquito pools. After being captured and brought to the lab, mosquitoes were identified at the species level using common taxonomy characteristics [17]. From August 2019 to September 2021, adult female *Aedes* mosquitoes were gathered from ten distinct places in Bhopal’s wards no. 52–61. All of the female mosquitoes were separated into 40 pools (15–18 mosquitoes per pool) and stored at −80 °C until further use. Each mosquito pool received 1.5 mL of 4% bovine serum albumin in phosphate buffer saline (PBS, pH 7.8) at the time of processing, using a pestle and mortar (Figure 2) [18].

### 2.5. In-Vitro Culture of Dengue Virus in Vero E6 Cell Line

Vero-E6 cells were maintained in MEM media containing 10% FBS, and viral infection in Vero cells was performed using a method mentioned in a previous study [19]. Briefly, 100 µL of liquid from mosquito processing was added to 70% confluent cultured Vero cells. After CPE was observed, the virus-containing cell culture supernatant was kept at −80 °C until the RNA extraction, cDNA conversion, and qRT PCR.

### 2.6. Establishing the Positive Control and qRT-PCR for Detection of Dengue Virus

PCR-positive clone generation was performed according to the method published previously [20]. Briefly, the cloning of the 112 base pair region of DENV-1 and 79 base pair region of DENV-2 was performed using the CloneJET PCR Cloning Kit (Thermo Fisher Scientific, Waltham, MA, USA) and T4 DNA ligase. The positive clones were then confirmed by colony PCR (Supplementary Figure S1). After confirmation, the cloned plasmids having the desired segment of dengue virus were isolated using a plasmid isolation kit (MACHEREY-NAGEL GmbH & Co. KG. NucleoSpin Plasmid Kit, REF 740588.50; Düren, Germany). Successive dilutions of the plasmid from 10^9^ to 10^2^ copies/mL were made after isolation.

Serotyping and quantification were performed using a commercial kit: Takyon Master Mix by Eurogentec (Eurogentec, Liège, Belgium; Cat.#UF-LPMT-B0701). Multiplexing qRT-PCR was performed to identify all four dengue virus serotypes (Figure 2) [21].

### 2.7. CPrM Region Sanger Sequencing and Whole Genome Sequencing of DENV Virus

From the serum samples that tested positive for NS1, total RNA was extracted using TRIzol reagent (Sigma-Aldrich, St. Louis, MO, USA; Cat. # T3934) according to the manufacturer’s protocol. Then, using an iScript cDNA synthesis kit (Bio-Rad Laboratories, Hercules, CA, USA; Cat. #1708897), 10 µL of the extracted RNA was converted into cDNA. DENV-specific RT-PCR primers were used to amplify the 511 base pair region of the CPrM gene [22]. This 511 bp of CPrM region was sequenced using the Sanger sequencing method (Supplementary Figure S2). The whole genome of the dengue virus was sequenced using Illumina sequencing by the method described previously (Figure 2) [23].

### 2.8. Phylogenetic Analysis of the CPrM Regions and the Whole Genome Sequences 

The sequence alignment was performed using the MUSCLE program incorporated into the MEGA 10 software [24]. The maximum-likelihood approach was used to build the phylogenetic tree for the CPrM regions as well as for the whole genome of all host and mosquito pool samples. A total of 1000 replicates were used to calculate the bootstrap values (Figure 2).

### 2.9. Shannon Entropy Analysis for the WGS of DENV Virus

HIV sequences database software (https://www.hiv.lanl.gov/cgi-bin/ENTROPY/entropy.cgi, accessed on 20 May 2024) was used to examine the Shannon entropy of DENV-1 and DENV-2. The probability of disorder or variation at a certain amino acid position in protein sequences is increased by the amino acid’s entropy [25]. The entropy graph was then created by manually editing values from an online software database into Microsoft Excel sheets.

### 2.10. Molecular Clock Analysis of the WGS Using Bayesian MCMC

Molecular clock analysis was performed using a dataset that included eight whole genome sequences of DENV-2 (a total of sixty-two sequences) and two whole genome sequences of DENV-1 (a total of fifty-two sequences) from this investigation. The Bayesian MCMC approach, provided in BEAST v1.8.4 [26], was used to calculate the nucleotide substitution rates and time to their most recent common ancestor (TMRCA) of the DENV-1 and DENV-2 strains. The Bayesian information criterion (BIC) was used to determine the best-suited nucleotide substitution model using jModelTest 2 [27]. Based on the lowest BIC (Bayesian Information Criterion) scores, the GTR + G model for DENV-1 and the TrN93 + G + I model for DENV-2 were determined to be the most appropriate models for Bayesian Markov Chain Monte Carlo (MCMC) analysis. We employed a strict molecular clock model with constant size for both viruses. The MCMC chain was run in two stages: 100,000 for DENV-1 and 30,000 for DENV-2. Every 1000 steps, parameter values were sampled. Log-Combiner v1.8.1 was used to merge the log files from the two different MCMC investigations, with 10% burn-in sets for each run. Log data were viewed using Tracer v1.6 in order to confirm chain convergence and ascertain whether an effective sample size (>200) had been achieved for all parameters. HPD intervals of 95% were assessed to ascertain the degree of uncertainty in the parameter estimates. TreeAnnotator v1.8.1 was used to generate a maximum clade credibility tree. Figtree v1.4.2 was used to display the generated tree file.

### 2.11. Neutralizing Antibody Assay for Control Samples

Serum samples of 67 apparently healthy volunteers from wards No. 52–61 and contacts of dengue patients staying in the same household who came into the AIIMS OPD for routine checkups were studied for DENV-neutralizing antibodies (Figure 2). For this, indirect DENV IgG enzyme-linked immunosorbent assay (IgG-ELISA) was performed using a recombinant DENV envelope (Domain-III) for serotype 1, 2, 3 and 4 with specific antigens (DEN-005 for DENV-1, DEN-007 for DENV-2, and DEN-008 for DENV-3, and DEN-009 for DENV-4) used (ProSpec-Tany TechnoGene Ltd., Ness Ziona, Israel). Each antigen was used as 1 µg/well (1 µg/100 µL/well). The neutralization test was performed for in-vitro viral inhibition (cytopathic effect inhibition) with some modification [28]. All 67 heat-inactivated human serum samples were diluted 1:20 in MEM (2% heat-inactivated FBS) and incubated with DENV in a CO_2_ incubator at 37 °C for one hour. This mixture was added to the cells, and the 96-well plates were incubated at 37 °C for five days. The test was performed with the following setups: (i) Vero cells without DENV, (ii) Vero cells with DENV served as a positive control (DENV 1–4), and (iii) Vero cells with the mixture (virus and diluted serum from the healthy individual) (Supplementary Figure S3).

## 3. Results

**Figure 3:**
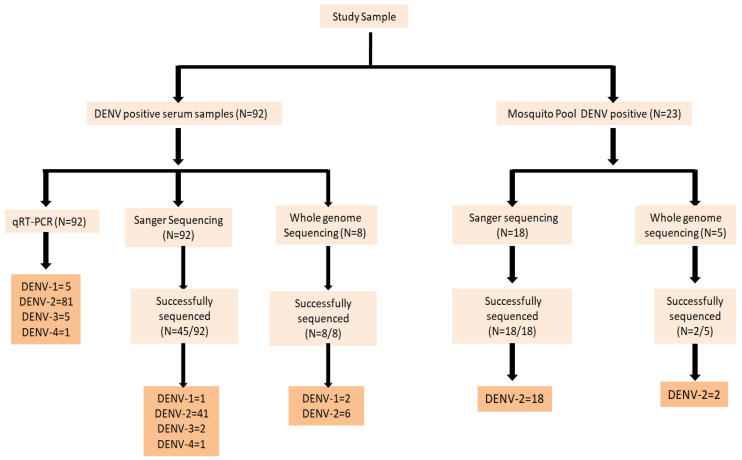
Details of samples used in this study.

From a total of 280 serum samples collected from suspected dengue patients, 92 samples tested positive for NS1. Of these positive cases, the CPrM gene was successfully sequenced in 45 samples using the Sanger sequencing method, while 8 samples underwent whole genome sequencing (WGS) (Figure 3).

The clinical profiles of suspected DENV cases, including those who tested NS1 negative, revealed that patients ranged in age from 18 to 65 years, encompassing both males and females. Common symptoms reported were low to high-grade fever persisting for 5–6 days, along with myalgia, arthralgia, thrombocytopenia in approximately 19–20% of cases, and abnormal liver function tests (LFT) (Table 1).

A total of 735 adult female *Aedes* mosquitoes (532 *Ae. aegypti* and 203 *Ae. albopictus*) were collected from wards of Bhopal, representing a population of 7–8 thousand per ward of the study area. A total of 40 pools of vector were processed, and among them, 23 pools from cell culture supernatant were positive for DENV RNA based on qRT-PCR (Supplementary Table S2). Eighteen pools were sequenced using Sanger sequencing for the CPrM gene, and two pools were sequenced using whole genome sequencing.

### 3.1. Serotype Specific qRT-PCR

**Figure 4:**
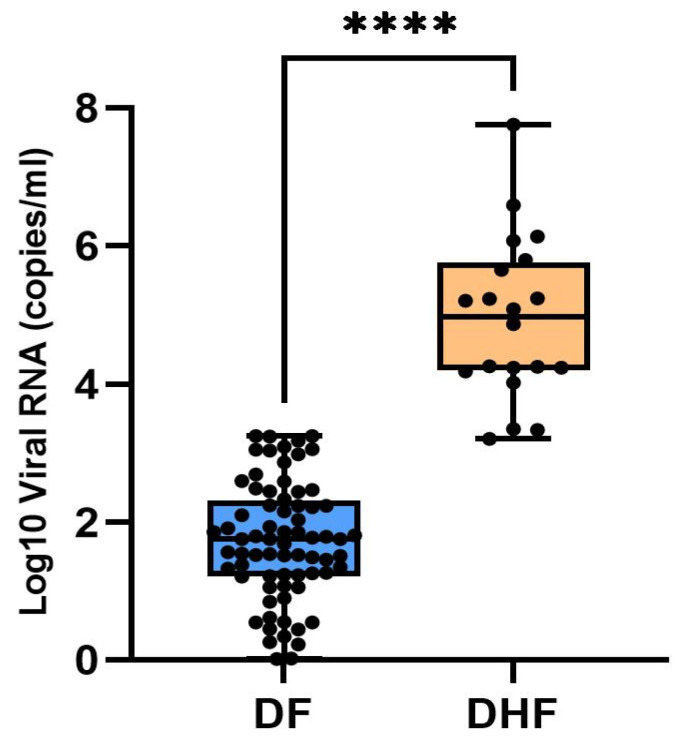
Viral load difference between DF and DHF patients (**** *p* < 0.0001).

qRT-PCR was conducted to determine the serotype and viral load of DENV in host samples collected during the study period. Viral load analysis showed a range of 0.2 to 7.7 Log viral RNA copies/mL in all 92 samples. Among the 20 patients of DHF or those requiring hospitalization, a significant difference (*p* < 0.0001) was observed in the average viral load, which was 4.93 Log_10_ viral RNA copies/mL, while the remaining 72 samples of DF had an average of 1.72 Log_10_ viral RNA copies/mL (Figure 4 and Supplementary Table S3)

### 3.2. Phylogenetic Analysis

#### 3.2.1. DENV Genotype Distribution for Vector Pool

**Figure 5:**
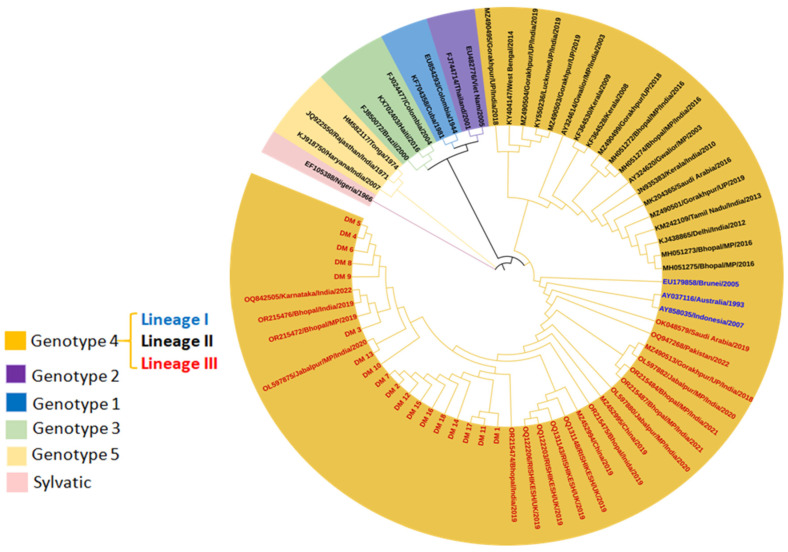
Phylogenetic tree based on CPrM gene of DENV-2 from a vector (*n* = 18) generated using Sanger sequencing. Each strain is identified by its GenBank accession number, country/state/city of origin, and the year of isolation. The analysis of DENV 2 was performed with the study isolates using the maximum likelihood and Tamura–Nei method in MEGA 10 software (Bootstrap = 1000).

Phylogenetic analysis revealed that eighteen DENV CPrM gene sequences from mosquitoes generated using Sanger sequencing belonged to serotype 2 (Figure 5). Analysis of the representative serotype-2 CPrM gene sequences from India and other countries clearly showed that all 18 sequences were clustered with non-Indian lineage of DENV-2 (Cosmopolitan genotype 4, lineage III). Other isolates obtained from Bhopal in 2016 (GenBank accession nos. MH051272-MH051275) were grouped into distinct clades within the most prevalent cosmopolitan 4 genotype lineage II.

#### 3.2.2. DENV Genotype Distribution for Host Serum Samples

Sequencing of 45 samples for the CPrM gene shows 1 patient was infected with DENV-1, 41 with DENV-2, 2 patients with DENV-3, and 1 with DENV-4. Although we found that all serotypes were in circulation in Bhopal from September 2019 to November 2023, among them DENV-2 was the predominant serotype with 41 isolates.

To determine the genotype distribution of DENV within each serotype, CPrM gene sequences obtained during this study and sequences from different geographical locations across the globe were retrieved from the NCBI database, aligned, and used for phylogenetic analyses.

Phylogenetic analysis identified one sequence from this study, generated through Sanger sequencing, as belonging to DENV-1 and classified it within clade C of genotype 5 (refer to Supplementary Figure S4). Notably, this finding aligns with previous observations of isolates from 2016 circulating in Bhopal (MH051267-MH051271), which also belonged to the same clade. Additional members of this genotype were traced to sequences originating from Thailand (1980), Singapore (2004–2015), and China (2014).

**Figure 6:**
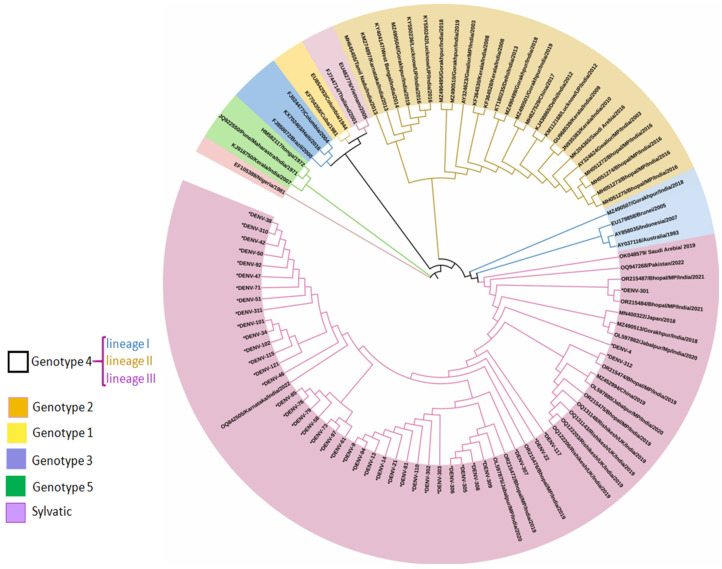
Phylogenetic tree-based CPrM gene of DENV-2 from hosts (*n* = 41) generated using Sanger sequencing. Each strain is identified by its GenBank accession number, country/state/city of origin, and the year of isolation. The analysis of DENV 2 was performed with the study isolates by using the maximum likelihood and Tamura–Nei method in MEGA 10 software (Bootstrap = 1000).

As evident, DENV-2 sequences were categorized into six genotypes [29]. The studied isolates generated using Sanger sequencing during the period of September 2019 to November 2023 (*n* = 41) were classified as Cosmopolitan or genotype 4 (lineage III) (Figure 6). This genotype contains three geographically distinct lineages: lineage I (Australian, Indonesian, Brunei), lineage II (isolates from Southeast Asia, China, and Oceania), and lineage III (isolates mostly from the Indian subcontinent). Present DENV-2 sequences from Bhopal were attributed to lineage III, clustering mostly with Indian strains. Conversely, other isolates (OR215476, OR215472, OL597875, OR215475, OR215474, OL597880, OL597882, OR215487, and OR215484) were also circulated in the same region and grouped in the same lineage (lineage III), while isolates from 2016 (MH051272-MH051275) were clustered in genotype lineage II.

Phylogenetic analysis demonstrated that the study isolates which were generated using Sanger sequencing were clustered within genotype III of DENV-3 (*n* = 2) (Supplementary Figure S5). Genotype III was further subdivided into five lineages. The current DENV-3 sequences were closely related to isolates from India (2008–2019), China (2013), and Singapore (2009, 2012). The study isolates from 2019 to 2021 were clustered within lineage III, similar to previously reported isolates from 2016 in Bhopal (MH051276, MH051277) and other isolates from Madhya Pradesh (MK829114, MK829116, AY770511).

DENV-4, a rare serotype in India, was detected in the early 1960s in Vellore, Tamil Nadu [30], and responsible for a few outbreaks in Kanpur (1968–1969) [31] and Andhra Pradesh (2007) [32]. Serotype 4 is divided into five genotypes, and only study isolates from Bhopal were clustered within genotype I (Supplementary Figure S6), alongside isolates from other Asian countries (Sri Lanka, Thailand, Singapore, Cambodia, China, Pakistan, and the Philippines).

#### 3.2.3. Whole Genome Sequence Analysis

##### Phylogenetic Analysis

To assess the above observations based on partial sequencing (CPrM gene), some representative samples were subjected to whole genome sequencing (Illumina platform) for the in-depth study. Phylogenetic analysis was performed separately for both serotypes (DENV-1 and DENV-2), and the respective reference sequences were retrieved from GenBank (KF289073 for DENV-1 and EU854293 for DENV-2). The clean reads of isolates (two sequences of DENV-1 and eight sequences of DENV-2; six were from human serum, and two were from the mosquito pool) were aligned to the reference sequences. All isolates have a full-length sequence of 10,138 (DENV-1) and 10,136 (DENV-2) nucleotides, which were used for the phylogenetic study. Similar results were found from whole genome sequences of DENV-1 (*n* = 2) belonging to the clade C of genotype V isolated from Bhopal during 2019–2023 (Supplementary Figure S7); the DENV-2 sequences from Bhopal during the period of 2019–2023 (*n* = 8) were clustered within genotype IV (Lineage III) (Supplementary Figure S8).

##### Shannon Entropy Analysis

**Figure 7:**
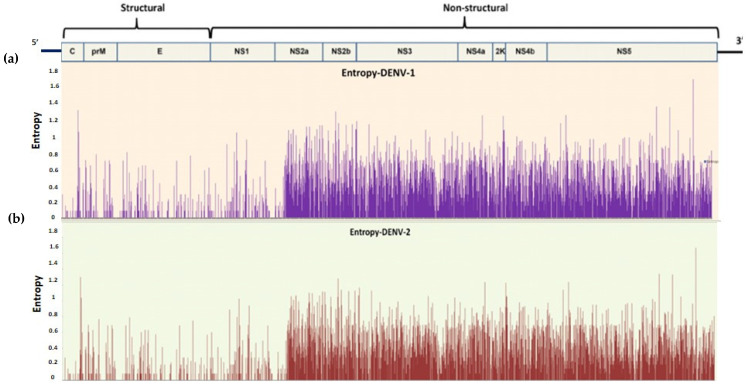
Shannon entropy plot of the whole proteome of dengue virus. (**a**) DENV-1 and (**b**) DENV-2.

A relatively high degree of intra-type and inter-type sequence conservation was observed, with low entropy values, generally below on average 0.25, indicating a relatively low level of viral evolution and diversity [33]. The identification of the sites that are prone to mutation in the whole proteome of the DENV was performed for the datasets with *n* = 58 (*n* = 2 from the present study with 56 sequences retrieved from NCBI) (DENV-1) and *n* = 62 (*n* = 8 from the present study and 54 sequences retrieved from NCBI) (DENV-2). For the selection of a variable site, a value of 0.2 was set as the threshold. The inter-type entropy difference between both the serotypes is relatively not so large (±0.05). However, inter-type all structural proteins are relatively conserved with low entropy value (capsid~0.04, PrM~0.067, Envelop~0.04, NS1~0.05), while all the non-structural proteins were showing substantial divergence with relatively high entropy values (NS2A~0.413, NS2B~0.37, NS3~0.35, NS4A~0.35, 2K peptide~0.43, NS4B~0.34, and NS5~0.33) (Figure 7).

##### Bayesian MCMC Analysis

**Figure 8:**
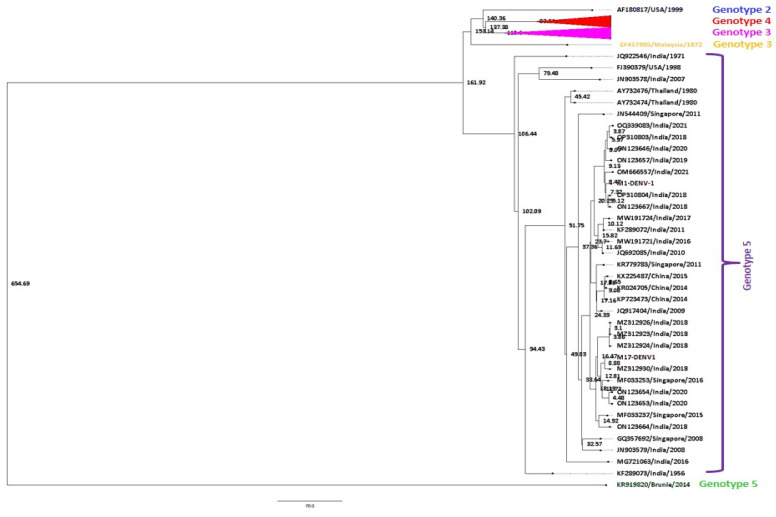
Maximum clade credibility tree of DENV-1 (*n* = 58). The tree was generated with the best-fit strict clock, the Bayesian skyline model. Node ages are denoted at each node.

The best-fit nucleotide substitution model for the DENV-1 (*n* = 58) dataset includes all of its six genotypes. The best-fit nucleotide substitution model for the dataset was chosen as GTR + G (gamma categories = 4). A strict constant size clock tree prior was chosen as the best-fit model in the molecular clock analysis. The maximum clade credibility (MCC) tree for DENV-1 sequences was generated in Fig tree v1.4.1 using the best-fit model (Figure 8).

The mean nucleotide substitution rate under the strict clock was detected to be 3.77 × 10^−4^ substitutions per site per year (95% HPD (3.40 × 10^−4^ to 4.12 × 10^−4^ s/s/y)). The age from the root was estimated to be approximately 654 years (95% HPD (582–735 years)), 1366 (95% HPD (1287–1337)). Similarly, the time to the most recent common ancestor (tMRCA) for genotype 5 was about 161 years (95% HPD (152–175 years)). Further, the study strains of DENV-1 clustered in genotype 4 were found to be approximately 8 years old (95% HPD (6–10 years)). Likewise, tMRCA for genotypes 2, 4, 3, and 1 was estimated to be around 140, 83, 153, and 137 years old, respectively.

**Figure 9:**
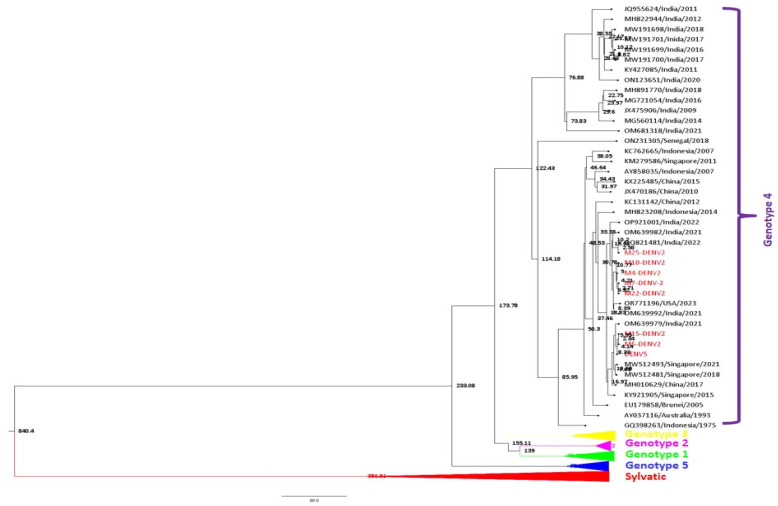
Maximum clade credibility tree of DENV-2 (*n* = 62). The tree was generated with the best-fit strict clock and constant size model. Node ages are denoted at each node.

The best-fit nucleotide substitution model for the dataset for DENV-2 (*n* = 62) was chosen as TrN 93 + I + G (gamma categories = 4). A strict constant size clock tree prior was chosen as the best-fit model in the molecular clock analysis. The maximum clade credibility (MCC) tree of DENV-2 sequences was generated in Fig tree v1.4.1 using the best-fit model (Figure 9).

A total of 3.62 × 10^−4^ substitutions per site per year (95% HPD (2.68 × 10^−4^ to 4.69 × 10^−4^ s/s/y)). The age from the root was estimated to be approximately 841 years (95% HPD (731–981 years)), 1180 (95% HPD (1040–1290)). Similarly, the time to the most recent common ancestor (tMRCA) for genotype 4 was about 174 years (95% HPD (156–191 years)). Further, the study strains of DENV-1 clustered in genotype 4 were found to be approximately 8 years old (95% HPD (6–10 years)). Likewise, tMRCA for genotypes 5, 1, 2, and 3 was estimated around 76, 80, 35, and 69 years, respectively.

### 3.3. Analysis of Neutralizing Antibody Assay for Control Samples

**Figure 10:**
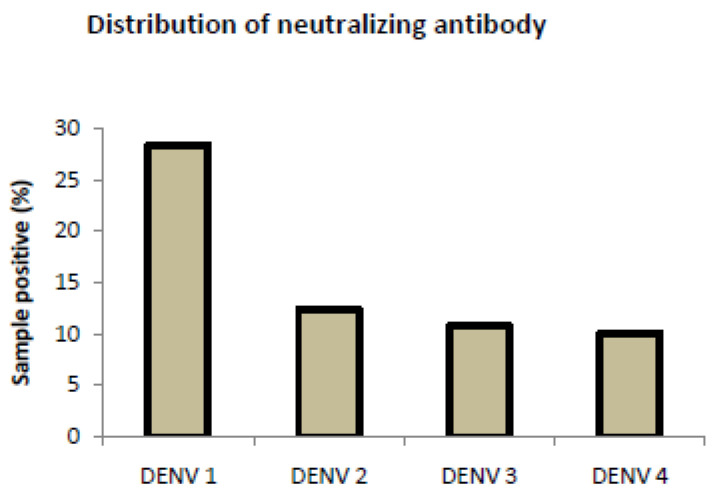
Plot representing the distribution of neutralizing antibodies in the population.

To assess the seroprevalence of dengue antibodies, serotype-specific ELISA was performed, followed by a neutralizing assay using Vero cell lines to observe the inhibition of CPE. The ELISA results revealed that 28.4% of healthy controls had IgG antibodies for DENV-1 infection, followed by DENV-2 (12.4%), DENV-3 (10.8%), and (10.1%) (Figure 10). However, we also found heterotypic or multitypic infection in some of the samples. The results of inhibition of cytopathic effect (CPE) using the Vero cell line revealed a serotype heterogeneity with seroprevalence detected for DENV-1, DENV-2, DENV-3, and DENV-4 in the studied wards (Supplementary Figure S9).

## 4. Discussion

Bhopal, one of the largest cities in terms of both population and area in Central India, an endemic dengue zone, has seen frequent DENV outbreaks over the past 20 years [10,11,12]. Dengue outbreaks, occurring every 2–3 years, result in significant morbidity and mortality in the region. Despite extensive research on dengue, knowledge gaps remain in understanding the circulating DENV variants, transmission dynamics, and factors influencing disease severity. Understanding dengue’s etiology and progression requires monitoring of genomic alterations, primarily through the evolutionary dynamics of sequences. To address these gaps, this study utilized genomic sequencing, phylogenetic analysis, and Bayesian MCMC to investigate the molecular epidemiology of DENV in hosts and vectors. Additionally, DENV seroprevalence was assessed in a seemingly healthy population to understand disease dynamics.

The present investigation revealed that DENV-2 was predominant in both the host and vector, followed by DENV-1, DENV-3, and DENV-4. The DENV-2 isolates recovered belong to genotype-IV (lineage III), dominating both host and vector samples, indicating an epidemiological relationship between DENV serotype mosquitoes and human cases. This study revealed a displacement of the native DENV-1 genotype-V (clade C) strain by the DENV-2 genotype-IV (lineage-III) strain in the Bhopal region. This transition was notable during the periods of 2016–2017 and 2019–2021, reflecting a competitive replacement between the serotypes (supplementary Figure S10). The new genotypic and lineage variants of DENV-2 circulating in the vectors may likely contribute to the higher incidence of dengue cases. According to earlier research, “stochastic events attributable to the low rate of virus transmission during the inter-epidemic period” could be the cause of the rise of dominant serotype [34]. Experimental infection trials within *Aedes aegypti* infected with either DENV-1, DENV-4 (mono-infection), or both viruses (co-infection) indicate that DENV-4 had a competitive advantage in a co-infection scenario. Although, no significant differences in transmission or dissemination were observed [35]. These findings emphasize the ability of mosquitoes in endemic areas to harbor multiple DENV serotypes, with natural selection driving the replacement of one genotype by another due to vector-mediated dynamics [36].

This study suggests that the DENV-2 serotype likely achieved greater viral loads in vectors compared to native DENV-1, enabling its rapid proliferation and dominance in the region. Seroprevalence statistics suggested that immunity against DENV-1 in the population provided limited protection against the newly emerged DENV-2 genotype. This lack of neutralizing antibodies against DENV-2 facilitated the outbreak.

The comparison of the viral loads (viremia) in host patients revealed an additional finding that the DENV NS1-positive participants who needed hospitalization for thrombocytopenia, high-grade fever, or abnormal liver function tests had high viral loads. These results are consistent with research from Vietnam showing that elevated viremia is linked to severe dengue manifestations such as DHF or DSS [15]. However, contrasting results from another study have been observed showing that viremia in DENV infection is not associated with a cytokine storm [16]. The findings suggest that a high viral load or reproduction number (R0) plays a crucial role in establishing a transmission chain, as it enables the host to maintain elevated viral loads early in the infection; these conditions facilitate the displacement of native strains and the propagation of new DENV genotypes. When mosquitoes feed on infected hosts with high viremia, it enables the transmission of emerging strains. This process underscores the epidemiological impact of viremia in driving the emergence and spread of novel DENV variants. The phenomenon of genetic variants and the competitive displacement of the original strain of the virus have had a substantial impact on the epidemiology and global pathogenicity of dengue illness [35,37].

Genetic analysis found a predominance of synonymous amino acid mutations, which do not alter the protein structure or function, suggesting sequence conservation across the viral proteome. This is further supported by the Shannon entropy analysis, indicating minimal variation in protein-coding regions. This stability has implications for vaccine development, as monitoring proteomic changes can help predict viral behavior and inform vaccine design strategies [38]. This study also signifies the use of non-structural genes as similar diversity to structural DENV genes; this may advocate distinct evolutionary pressures [39].

Bayesian evolutionary analysis estimated that DENV-1 and DENV-2 have their respective average nucleotide substitution rates of 3.77 × 10^−4^ substitutions per site year and 3.62 × 10^−4^ substitutions per site per year, respectively. This suggested that DENV-2 evolved more slowly than DENV-1 and experienced more early evolutionary events. The estimated mean root age of DENV-2 was 841 years (95% HPD 731–981 years), while the inferred mean root age of DENV-1 in 2021 was 654 years (95% HPD 582–735 years). These results are in line with research that has already been published [4,40]. It is mentioned that the earliest known cases of an illness similar to dengue fever were recorded in 992 during the Chin dynasty in China, which lasted from the third to the fifth century [41]. This year is also rather near to our mean estimate for the TMRCA of all serotypes. The broadest estimate suggests a composite TMRCA of DENV was about 1670 years ago [42]. This period corresponds with the first documented medical report of “water poison”, a dengue-like disease that was transmitted by mosquitoes in third-century China [26]. This study predicted a major divergence in cosmopolitan genotype stemmed from the emergence of an Indian subcontinent lineage approximately 123 years ago.

Vectors play a crucial role in the emergence of new genotypes, as viral replication in mosquitoes influences the evolutionary trajectory of DENV. This study also provides crucial information on how high viremia in hosts and the emergence of new DENV in vectors by natural selection maintain the transmission cycle in endemic areas. However, predicting the outbreak or emergence of a new DENV strain remains a difficult task despite advances in understanding the host–virus pathophysiology, tracing the evolution of the viruses, and genetic epidemiology. A key limitation of this study was the inability to evaluate differences between primary and secondary DENV infections, which could provide deeper insights into disease dynamics. Until the proper antiviral medication or vaccines are developed, routine surveillance of DENV serotypes and genotypes in endemic areas will remain essential for forecasting large-scale outbreaks. Early detection of shifts in circulating strains can inform public health interventions, minimizing the impact of dengue epidemics.

## 5. Conclusions

According to the current study, DENV-2 was the most prevalent serotype during the dengue outbreaks in the Bhopal region of Central India in 2019 and 2021, followed by other serotypes. Compared to earlier studies, tMRCA was found earlier, and low substitution rates based on the entire genome were noted. The current study investigated how the high viral load emerging DENV-2 genotype-IV (lineage-III) replaced the circulating native strain of DENV-1 and DENV-2, despite some disagreement on the origin time estimate of DENV. It may be brought on by the presence of monotypic protective antibodies against a particular serotype or by the competitive displacement of novel strains with high viral loads. In this endemic region, new serotypes and genotypes may emerge as a result of future outbreaks, as the study also found that all serotypes are co-circulated at the vector level. It is unknown which serotype will ultimately manifest in the vector’s midgut to initiate an outbreak. Molecular characterization, epidemiology, and vector surveillance, however, can greatly enhance predictions for severe dengue outbreaks in the future and break the cycle of outbreaks that recur every two to three years, which is linked to high death rates in the region. Continuous vector, virus, and DENV molecular epidemiology surveillance would help medical professionals make the necessary preparations for future outbreaks in the absence of a dengue vaccine in endemic areas.

## Figures and Tables

**Table 1:** Clinical history of DF and DHF/hospitalized dengue patients.

		NS1 ELISA Positive		NS-1 Negative (*n* = 188)
Clinical and Demographic Characteristics		DF * (*n* = 72)	DHF * (*n* = 20)	
Age (years)	18–30	23	6	88
	30–50	34	9	32
	50–65	15	5	68
Sex (Male/female)		45/27	15/5	110/78
Headache		12 (16%)	10 (50%)	56 (29%)
Myalgia		13 (18%)	20 (100%)	40 (21%)
Fever		65 (90%)	16 (80%)	165 (88%)
Arthralgia		10 (13%)	16 (80%)	89 (47%)
Rash		9 (12.5%)	4 (20%)	32 (17%)
Vomiting/Nausea		6 (8.33%)	3 (15%)	23 (12%)
Retro-orbital pain		5 (6.94%)	13 (65%)	15 (8%)
Hematocrit		14 (19%)	8 (40%)	25 (13%)
Thrombocytopenia		15 (20%)	18 (90%)	15 (8%)
Number of patients with increased aminotransferase level		32 (44%)	16 (80%)	8 (4%)

* DF = Dengue fever; * DHF = dengue hemorrhagic fever.

## Data Availability

Data is contained within the article or Supplementary Material. The Whole genome and Sanger sequenced sequences were submitted to NCBI (PP817678, PP817679, PP817680, PP817681, PP817682, PP817683, PP817684, PP817685, PP817686, PP817687, PP817688, PP817689, PP817690, PP817691, PP817692, PP817693, PP817694, PP817695, OR888734, OR888735, PP795941, PP795942, PP795943, PP795944, PP795945, PP795946, PP795947, PP795948, PP795949, PP795950, PP795951, PP795952, PP795953, PP795954, PP795955, PP795956, PP795957, PP795958, PP795959, PP795960, PP795961, PP795962, PP795963, PP795964, PP795965, PP795966, PP795967, PP795968, PP795969, PP795970, PP795971, PP795972, PP795973, OR492473, OR492474, OR492475, OR888730, OR888731, OR888732 OR888733, MZ841823, MZ841824, MZ841825, MZ841826, MZ841827, MZ841828, MZ841829,MZ841830).

## Supplementary Materials

The following supporting information can be downloaded at: https://www.mdpi.com/article/10.3390/v17020144/s1, Figure S1: 112 base pair band of dengue serotype 1 and 78 base pair band of dengue serotype 2 was cloned and confirmed on 2.5% agarose gel; Figure S2: 511 base pair band of dengue was confirmed on 1.5% agarose gel; Figure S3: Schematic representation of neutralizing antibody generation against DENV-1; Figure S4: Phylogenetic tree based on CPrM gene of DENV-1 from host (*n* = 1). Each strain is identified by its GenBank accession number, country/state/city of origin and the year of isolation. The analysis of DENV 1 was done with the Study isolates by using Maximum likelihood & Tamura-Nei method in MEGA 10 software (Bootstrap = 1000); Figure S5: Phylogenetic tree based on CPrM gene of DENV-3 from host (*n* = 2). Each strain is identified by its GenBank accession number, country/state/city of origin and the year of isolation. The analysis of DENV 3 was done with the Study isolates by using Maximum likelihood & Tamura-Nei method in MEGA 10 software (Bootstrap = 1000); Figure S6: Phylogenetic tree based on CPrM gene of DENV-4 from host (*n* = 1). Each strain is identified by its GenBank accession number, country/state/city of origin and the year of isolation. The analysis of DENV 4 was done with the Study isolates by using Maximum likelihood & Tamura-Nei method in MEGA 10 software (Bootstrap = 1000); Figure S7: Phylogenetic analysis of DENV-1 complete genome sequences (*n* = 2): Each strain is indicated by GenBank accession number followed by country and year of isolation. Numbers at the nodes are support values for the major branches (bootstrap; 1000 replicates). The sequences obtained in this study are marked in filled-colored circles. The scale bar indicates the number of base substitutions per site; Figure S8: Phylogenetic tree based on the whole genome of DENV-2 (*n* = 8). Each strain is identified by its GenBank accession number, country/state/city of origin and the year of isolation. The analysis of DENV 2 was done with the Study isolates by using Maximum likelihood & Tamura-Nei method in MEGA 10 software (Bootstrap = 1000); Figure S9: in vitro viral inhibition assay showing vero cell lines post infection of (Day 5) A. control cells, B. Vero cells infected with DENV-1 + DENV2 IgG antibody serum samples showing partial inhibition of viral growth, C. Vero cells lines infected with DENV-2 + DENV2 IgG antibody serum samples showing no inhibition of viral growth D. Vero cells infected with DENV3 + DENV2 IgG antibody serum samples showing partial inhibition of viral growth E. Vero cells infected with DENV-4 + DENV2IgGantibody serum samples showing complete inhibition of viral growth; Figure S10: Schematic representation of Dengue outbreak situation in Bhopal city during 2014–2016 and 2019–2021; Table S1: Patients’ clinical history at the time of OPD visit;. Table S2: Mosquitoes pools infected with different serotypes; Table S3: Serotype-specific viral load of DENV in host samples.

## Abbreviations

RC: Rashmi Chowdhary; AYK: Ashish Kumar Yadav; AK: Ashok Kumar; SK: Sagar Khadanga; RL: Rajnish Joshi; AP: Abhijit Pakhare; JRK: Jagar Rakesh Kanwar; AS: Arshi Siddiqui; SKG: Sudhir K Goel; DB: Debasis Biswas; AGM: Anvita Gupta Malhotra.

## References

[1] Bhatt S., Gething P.W., Brady O.J., Messina J.P., Farlow A.W., Moyes C.L., Drake J.M., Brownstein J.S., Hoen A.G., Sankoh O. (2013). The global distribution and burden of dengue. Nature.

[2] Li N.-K., Corander J., Grad Y.H., Chang H.-H. (2022). Discovering recent selection forces shaping the evolution of dengue viruses based on polymorphism data across geographic scales. Virus Evol..

[3] Rico-Hesse R. (1990). Molecular evolution and distribution of dengue viruses type 1 and 2 in nature. Virology.

[4] Holmes E.C., Twiddy S.S. (2003). The origin, emergence and evolutionary genetics of dengue virus. Infect. Genet. Evol..

[5] Putri D.H., Sudiro T.M., Yunita R., Jaya U.A., Dewi B.E., Sjatha F., Konishi E., Hotta H., Sudarmono P. (2015). Immunogenicity of a Candidate DNA Vaccine Based on the *prM/E* Genes of a Dengue Type 2 Virus Cosmopolitan Genotype Strain. Jpn. J. Infect. Dis..

[6] Chen R., Vasilakis N. (2011). Dengue—Quo tu et quo vadis?. Viruses.

[7] Bäck A.T., Lundkvist A. (2013). Dengue viruses—An overview. Infect. Ecol. Epidemiol..

[8] Heilman J.M., De Wolff J., Beards G.M., Basden B.J. (2014). Dengue fever: A Wikipedia clinical review. Open Med..

[9] Kularatne S.M., Ralapanawa U., Dalugama C., Jayasinghe J., Rupasinghe S., Kumarihamy P. (2018). Series of 10 dengue fever cases with unusual presentations and complications in Sri Lanka: A single centre experience in 2016. BMC Infect. Dis..

[10] Agarwal A., Ganvir R., Kale D., Chaurasia D., Kapoor G. (2024). Continued dominance of dengue virus serotype 2 during the recent Central India outbreaks (2019-2021) with evidence of genetic divergence. Pathog. Glob. Health.

[11] Pakhare A., Sabde Y., Joshi A., Jain R., Kokane A., Joshi R. (2016). A study of spatial and meteorological determinants of dengue outbreak in Bhopal City in 2014. J. Vector Borne Dis..

[12] Baruah K., Singh P., Mohalia M., Dhariwal A. (2010). A study on dengue outbreak during 2009 in Bhopal and Indore districts of Madhya Pradesh, India. J. Commun. Dis..

[13] Jagtap S., Pattabiraman C., Sankaradoss A., Krishna S., Roy R. (2023). Evolutionary dynamics of dengue virus in India. PLOS Pathog..

[14] Guzman M.G., Alvarez M., Rodriguez-Roche R., Bernardo L., Montes T., Vazquez S., Morier L., Alvarez A., Gould E.A., Kourí G. (2007). Neutralizing Antibodies after Infection with Dengue 1 Virus. Emerg. Infect. Dis..

[15] Vuong N.L., Quyen N.T., Tien N.T., Tuan N.M., Kien D.T., Lam P.K., Tam D.T., Van Ngoc T., Yacoub S., Jaenisch T. (2021). Higher plasma viremia in the febrile phase is associated with adverse dengue outcomes irrespective of infecting serotype or host immune status: An analysis of 5642 Vietnamese cases. Clin. Infect. Dis..

[16] de Arruda T.B., Bavia L., Mosimann A.L.P., Aoki M.N., Sarzi M.L., Conchon-Costa I., Wowk P.F., Duarte dos Santos C.N., Pavanelli W.R., Silveira G.F. (2023). Viremia and Inflammatory Cytokines in Dengue: Interleukin-2 as a Biomarker of Infection, and Interferon-α and -γ as Markers of Primary versus Secondary Infection. Pathogens.

[17] Tyagi B.K., Munirathinam A., Venkatesh A. (2015). A catalogue of Indian mosquitoes. Int. J. Mosq. Res..

[18] de Figueiredo M.L., de C Gomes A., Amarilla A.A., de S Leandro A., de S Orrico A., de Araujo R.F., do SM Castro J., Durigon E.L., Aquino V.H., Figueiredo L.T. (2010). Mosquitoes infected with dengue viruses in Brazil. Virol. J..

[19] Armstrong P.M., Andreadis T.G., Finan S.L., Shepard J.J., Thomas M.C. (2011). Detection of Infectious Virus from Field-collected Mosquitoes by Vero Cell Culture Assay. J. Vis. Exp. JoVE.

[20] Prada-Arismendy J., Castellanos J.E. (2011). Real time PCR. Application in dengue studies. Colomb. Medica.

[21] Santiago G.A., Vergne E., Quiles Y., Cosme J., Vazquez J., Medina J.F., Medina F., Colón C., Margolis H., Muñoz-Jordán J.L. (2013). Analytical and Clinical Performance of the CDC Real Time RT-PCR Assay for Detection and Typing of Dengue Virus. PLoS Negl. Trop. Dis..

[22] Lanciotti R.S., Calisher C.H., Gubler D.J., Chang G.J., Vorndam A.V. (1992). Rapid detection and typing of dengue viruses from clinical samples by using reverse transcriptase-polymerase chain reaction. J. Clin. Microbiol..

[23] Cruz C.D., Torre A., Troncos G., Lambrechts L., Leguia M. (2016). Targeted full-genome amplification and sequencing of dengue virus types 1–4 from South America. J. Virol. Methods.

[24] Kumar S., Stecher G., Li M., Knyaz C., Tamura K. (2018). MEGA X: Molecular Evolutionary Genetics Analysis across Computing Platforms. Mol. Biol. Evol..

[25] Pollett S., Melendrez M., Maljkovic Berry I., Duchêne S., Salje H., Cummings D., Jarman R. (2018). Understanding dengue virus evolution to support epidemic surveillance and counter-measure development. Infect. Genet. Evol. J. Mol. Epidemiol. Evol. Genet. Infect. Dis..

[26] Papastamoulis P., Furukawa T., Van Rhijn N., Bromley M., Bignell E., Rattray M. (2020). Bayesian detection of piecewise linear trends in replicated time-series with application to growth data modelling. Int. J. Biostat..

[27] Darriba D., Taboada G.L., Doallo R., Posada D. (2012). jModelTest 2: More models, new heuristics and parallel computing. Nat. Methods.

[28] Adam A., Schüttoff T., Reiche S., Jassoy C. (2018). High seroprevalence of dengue virus indicates that dengue virus infections are frequent in central and eastern Sudan. Trop. Med. Int. Health.

[29] Tiraki D., Singh K., Shrivastava S., Mishra A.C., Arankalle V. (2021). Complete genome characterization and evolutionary analysis of dengue viruses isolated during 2016–2017 in Pune, India. Infect. Genet. Evol..

[30] Gupta N., Srivastava S., Jain A., Chaturvedi U.C. (2012). Dengue in India. Indian J. Med. Res..

[31] Chaturvedi U.C., Mathur A., Kapoor A.K., Mehrotra N.K., Mehrotra R.M.L. (1970). Virological study of an epidemic of febrile illness with haemorrhagic manifestations at Kanpur, India, during 1968. Bull. World Health Organ..

[32] Dash P.K., Sharma S., Srivastava A., Santhosh S.R., Parida M.M., Neeraja M., Subbalaxmi M.V.S., Lakshmi V., RAO P.V.L. (2011). Emergence of dengue virus type 4 (genotype I) in India. Epidemiol. Infect..

[33] Islam A., Deeba F., Tarai B., Gupta E., Naqvi I.H., Abdullah M., Dohare R., Ahmed A., Almajhdi F.N., Hussain T. (2023). Global and local evolutionary dynamics of Dengue virus serotypes 1, 3, and 4. Epidemiol. Infect..

[34] Myat Thu H., Lowry K., Jiang L., Hlaing T., Holmes E.C., Aaskov J. (2005). Lineage extinction and replacement in dengue type 1 virus populations are due to stochastic events rather than to natural selection. Virology.

[35] Vazeille M., Gaborit P., Mousson L., Girod R., Failloux A.B. (2016). Competitive advantage of a dengue 4 virus when co-infecting the mosquito Aedes aegypti with a dengue 1 virus. BMC Infect. Dis..

[36] O’Connor O., Ou T.P., Aubry F., Dabo S., Russet S., Girault D., In S., Minier M., Lequime S., Home T. (2021). Potential role of vector-mediated natural selection in dengue virus genotype/lineage replacements in two epidemiologically contrasted settings. Emerg. Microbes Infect..

[37] Stica C.J., Barrero R.A., Murray R.Z., Devine G.J., Phillips M.J., Frentiu F.D. (2022). Global Evolutionary History and Dynamics of Dengue Viruses Inferred from Whole Genome Sequences. Viruses.

[38] Khan A.M., Hu Y., Miotto O., Thevasagayam N.M., Sukumaran R., Abd Raman H.S., Brusic V., Tan T.W., Thomas August J. (2017). Analysis of viral diversity for vaccine target discovery. BMC Med. Genom..

[39] Ali M., Pandey R.K., Khatoon N., Narula A., Mishra A., Prajapati V.K. (2017). Exploring dengue genome to construct a multi-epitope based subunit vaccine by utilizing immunoinformatics approach to battle against dengue infection. Sci. Rep..

[40] Twiddy S.S., Holmes E.C., Rambaut A. (2003). Inferring the Rate and Time-Scale of Dengue Virus Evolution. Mol. Biol. Evol..

[41] Gubler D.J. (1998). Dengue and Dengue Hemorrhagic Fever. Clin. Microbiol. Rev..

[42] Costa R.L., Voloch C.M., Schrago C.G. (2012). Comparative evolutionary epidemiology of dengue virus serotypes. Infect. Genet. Evol..

