# Supplementary material for: Emergence of a Novel Dengue Virus Serotype-2 Genotype IV Lineage III Strain and Displacement of Dengue Virus Serotype-1 in Central India (2019–2023)
Source: Viruses. 2025 Jan 23;17(2):144. doi: 10.3390/v17020144 (PMC11861835; doi:10.3390/v17020144)
Supplement: Supplementary file 1 [file viruses-17-00144-s001.zip › viruses-3371596-supplementary.pdf]

Supplementary Material

Colony PCR result

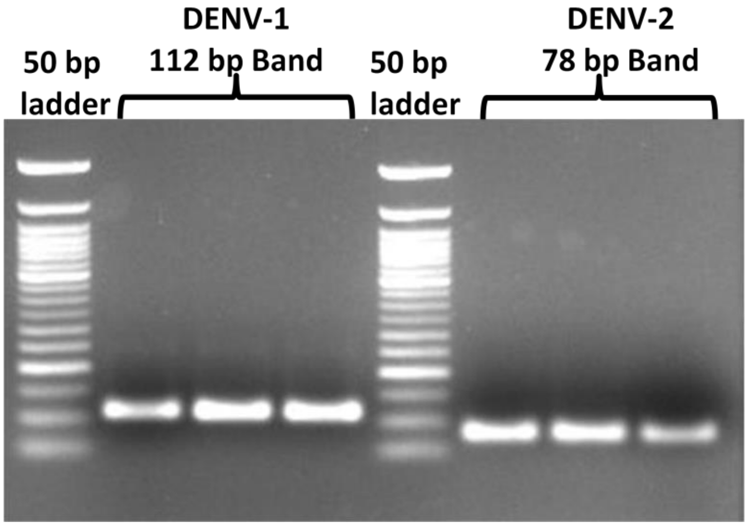

Supplementary Figure S1:112 base pair band of dengue serotype 1 and 78 base pair band of dengue serotype 2 was cloned and confirmed on 2.5% agarose gel.

CPrM gene amplification result

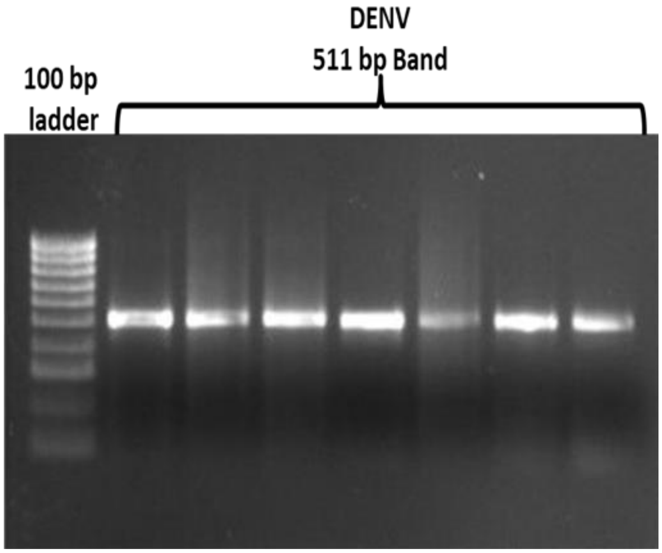

Supplementary Figure S2: 511 base pair band of dengue was confirmed on 1.5% agarose gel.

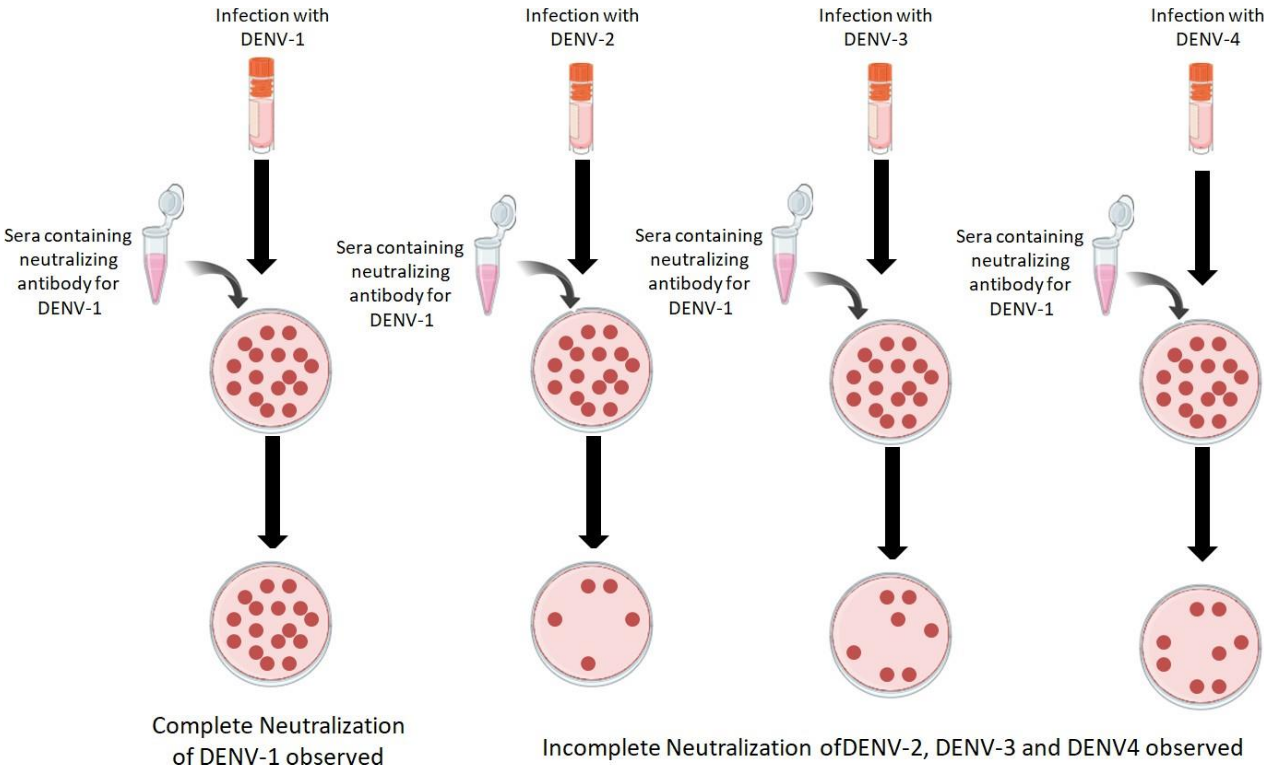

Supplementary figure 3: Schematic representation of neutralizing antibody generation against DENV-1.

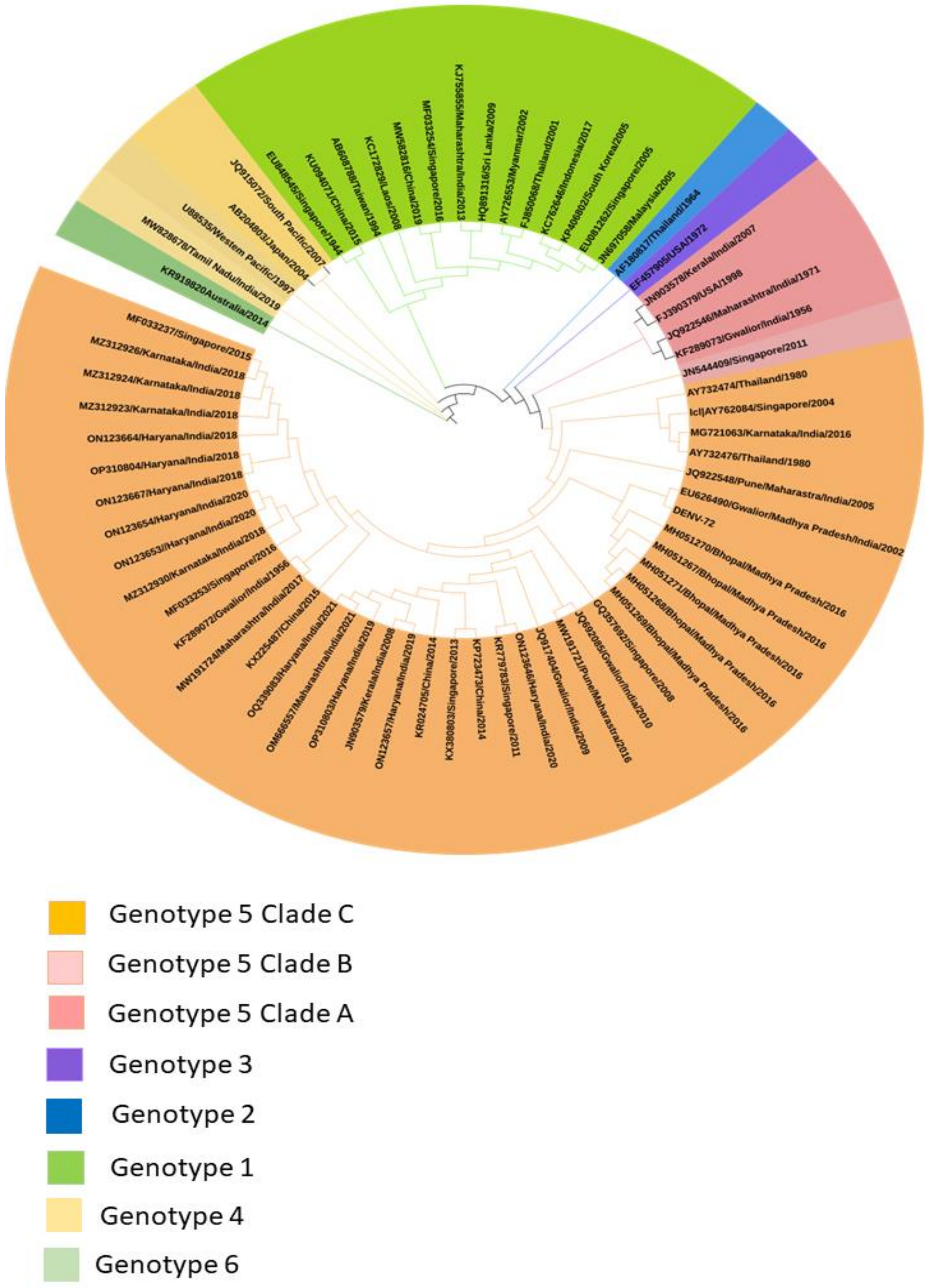

Supplementary figure S4: Phylogenetic tree based on CPrM gene of DENV-1 from host (n=1). Each strain is identified by its GenBank accession number, country/ state/city of origin and the year of isolation. The analysis of DENV 1 was done with the Study isolates by using Maximum likelihood & Tamura-Nei method in MEGA 10 software (Bootstrap=1000)

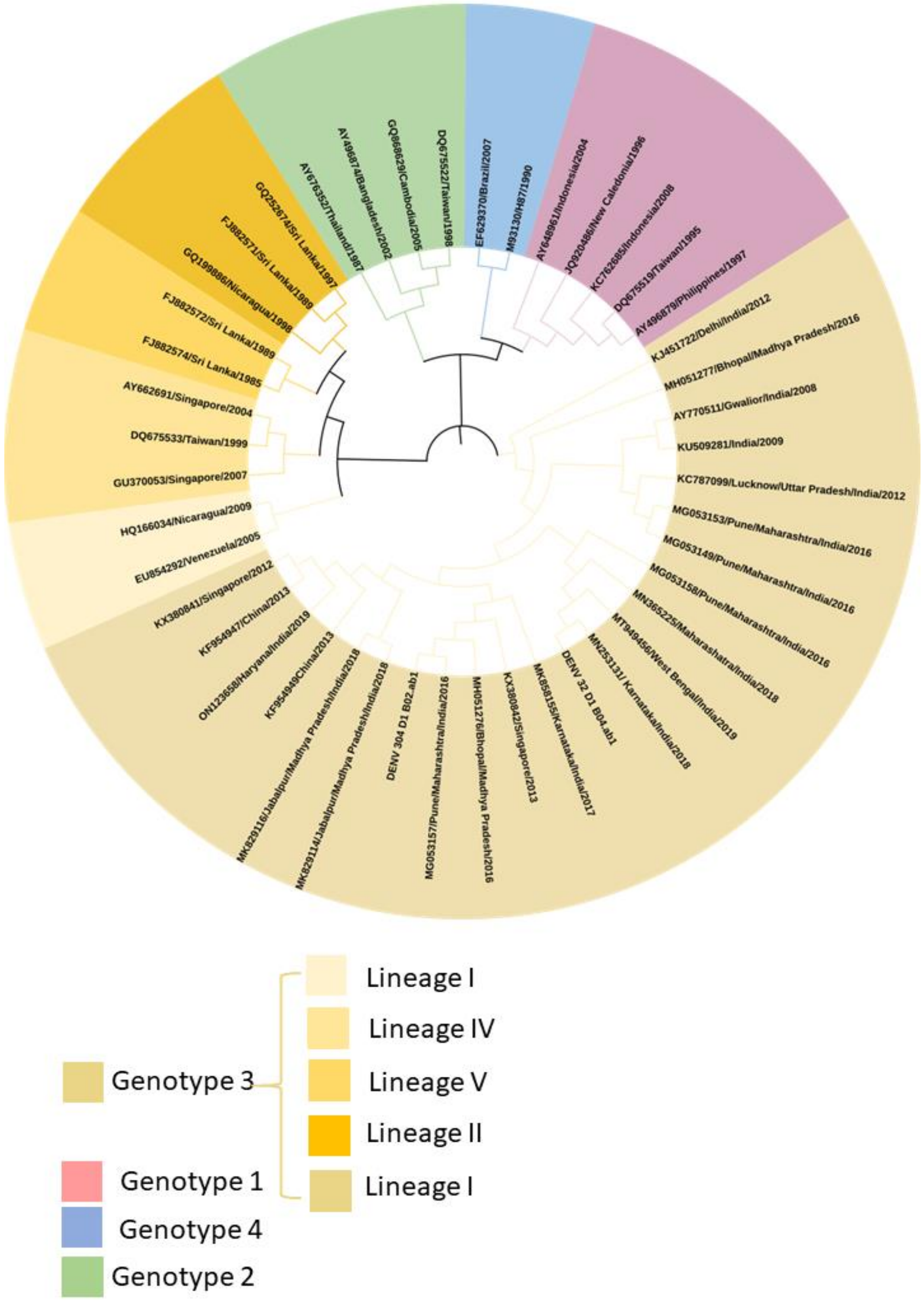

Supplementary figure S5: Phylogenetic tree based on CPrM gene of DENV-3 from host (n=2). Each strain is identified by its GenBank accession number, country/ state/city of origin and the year of isolation. The analysis of DENV 3 was done with the Study isolates by using Maximum likelihood & Tamura-Nei method in MEGA 10 software (Bootstrap=1000)

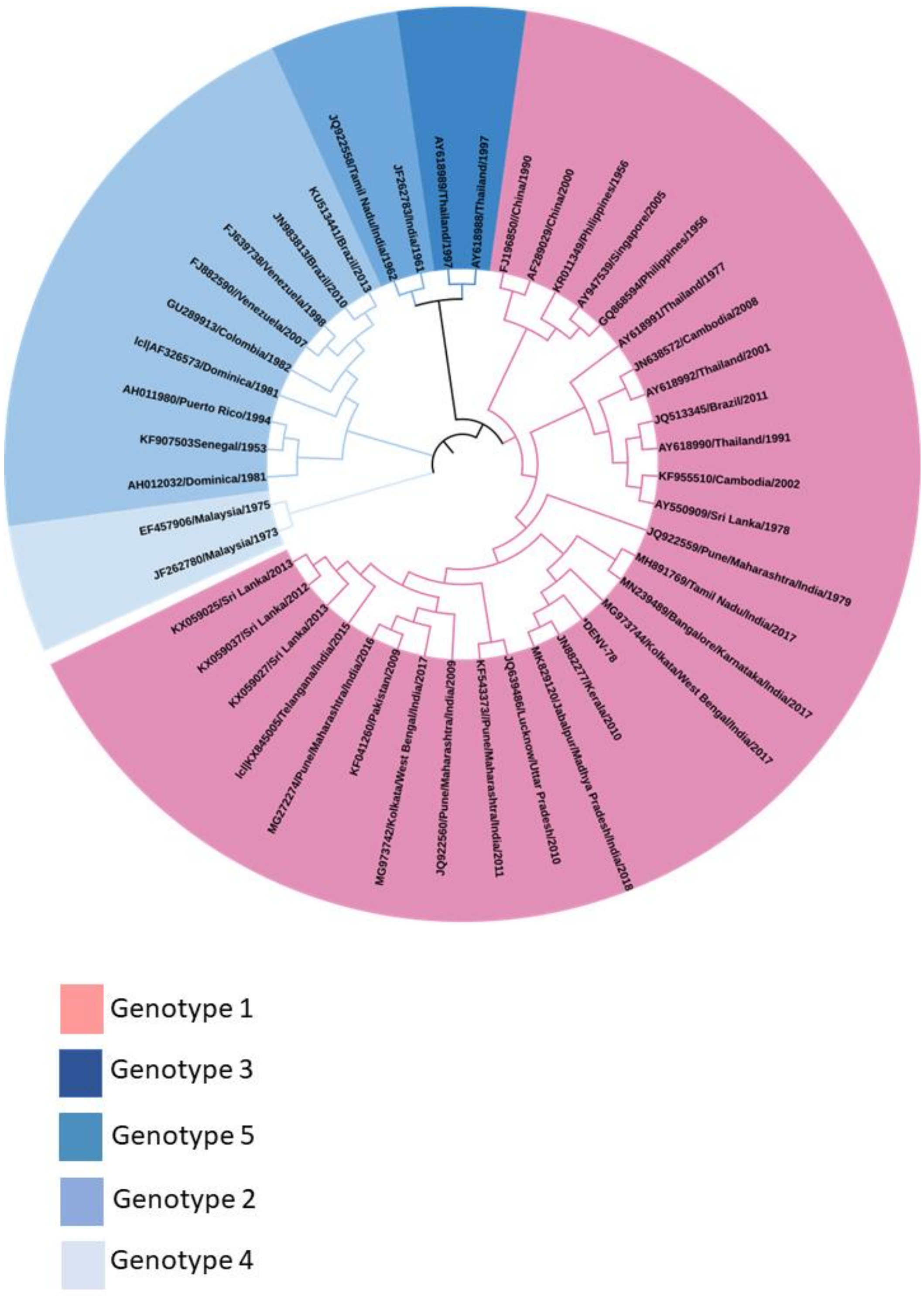

Supplementary figure S6: Phylogenetic tree based on CPrM gene of DENV-4 from host (n=1). Each strain is identified by its GenBank accession number, country/ state/city of origin and the year of isolation. The analysis of DENV 4 was done with the Study isolates by using Maximum likelihood & Tamura-Nei method in MEGA 10 software (Bootstrap=1000).

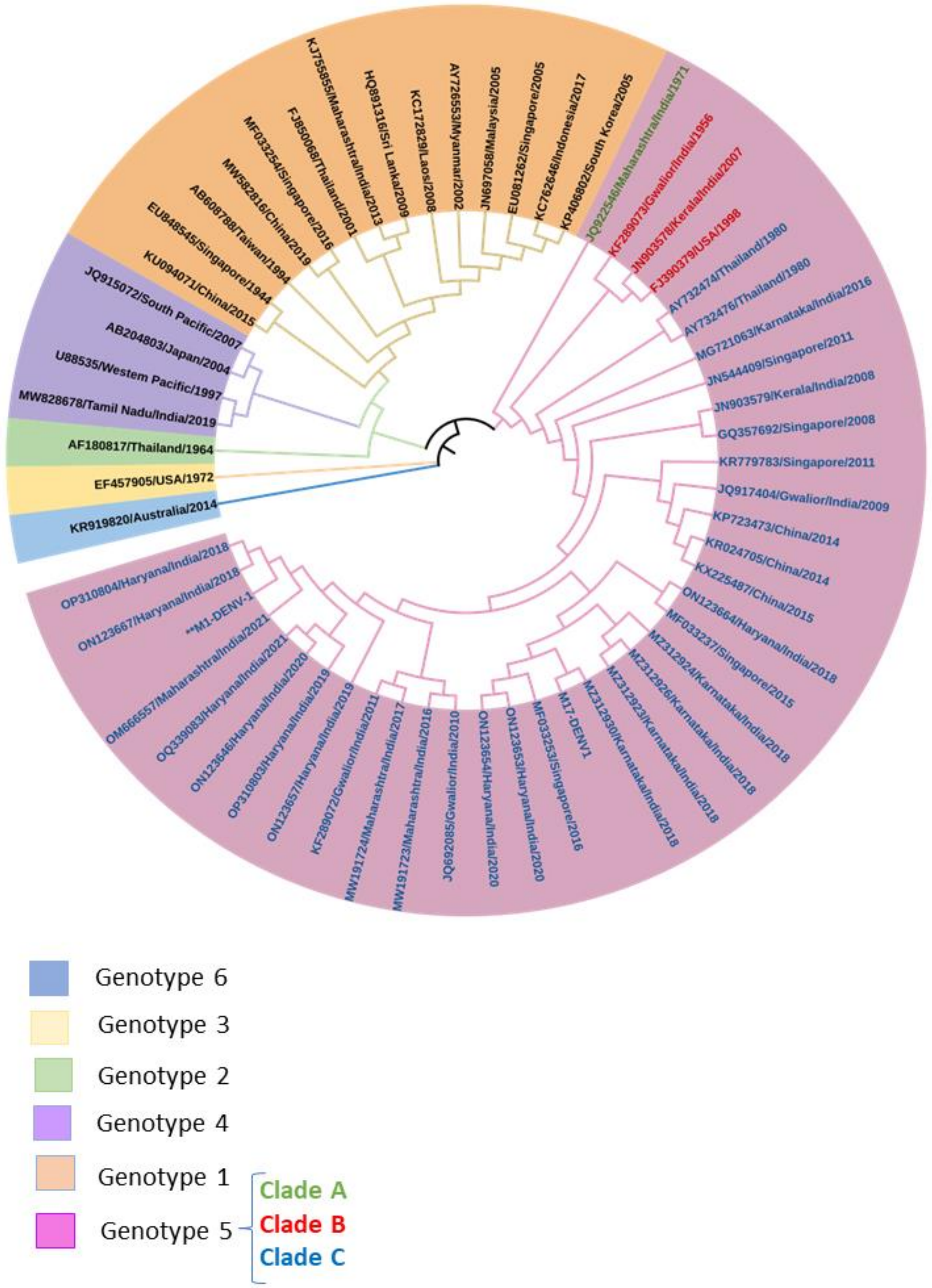

Supplementary figure S7: Phylogenetic analysis of DENV-1 complete genome sequences (n = 2): Each strain is indicated by GenBank accession number followed by country and year of isolation. Numbers at the nodes are support values for the major branches (bootstrap; 1000 replicates). The sequences obtained in this study are marked in filled-colored circles. The scale bar indicates the number of base substitutions per site.

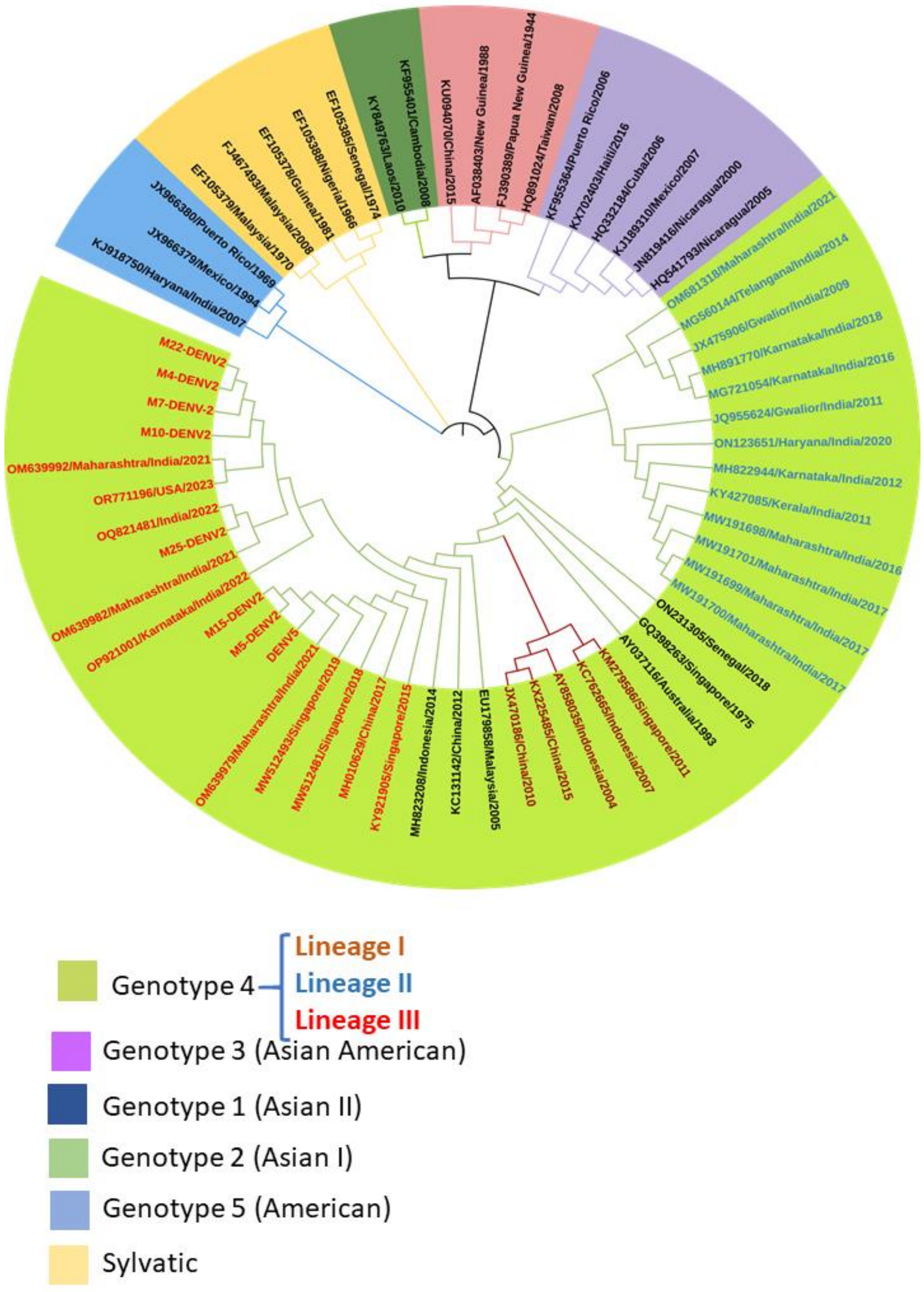

Supplementary figure S8: Phylogenetic tree based on the whole genome of DENV-2 (n=8). Each strain is identified by its GenBank accession number, country/ state/city of origin and the year of isolation. The analysis of DENV 2 was done with the Study isolates by using Maximum likelihood & Tamura-Nei method in MEGA 10 software (Bootstrap=1000)

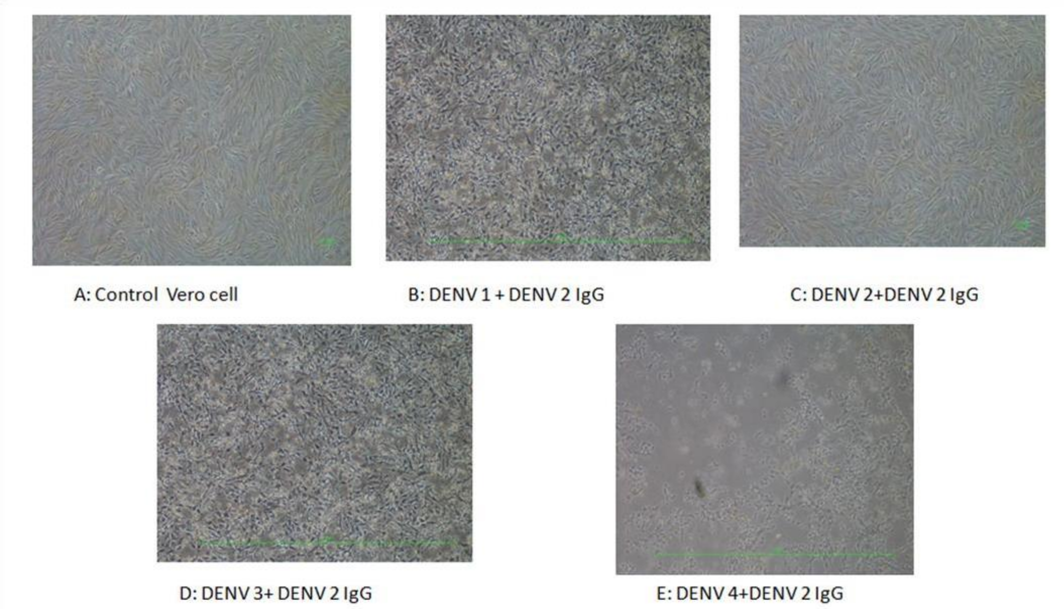

Supplementary Figure S9: in vitro viral inhibition assay showing vero cell lines post infection of (Day 5) A. control cells, B. Vero cells infected with DENV-1+ DENV2 IgG antibody serum samples showing partial inhibition of viral growth, C. Vero cells lines infected with DENV-2+ DENV2 IgG antibody serum samples showing no inhibition of viral growth D. Vero cells infected with DENV3+DENV2 IgG antibody serum samples showing partial inhibition of viral growth E. Vero cells infected with DENV-4+ DENV2IgGantibody serum samples showing complete inhibition of viral growth

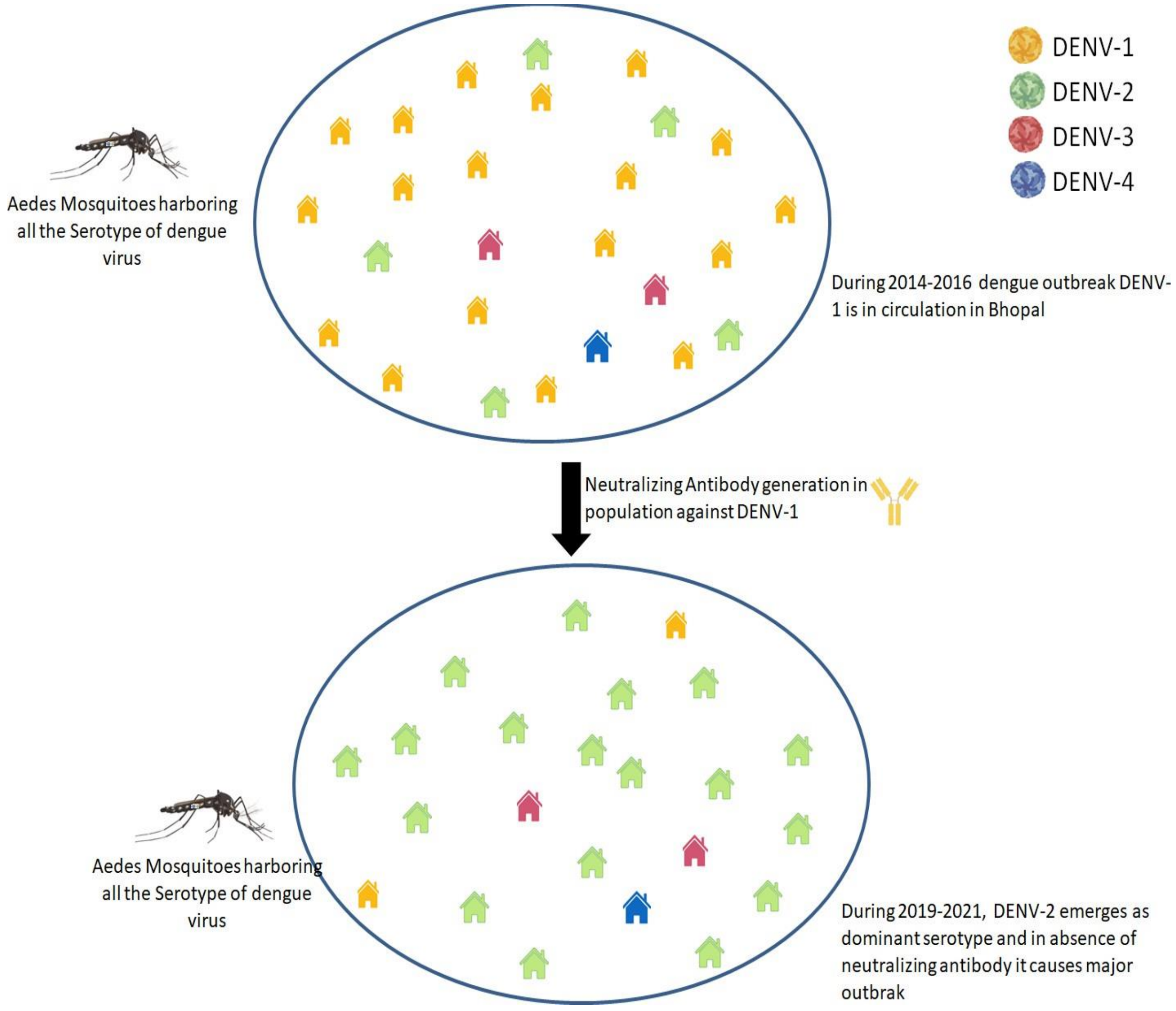

Supplementary figure S10: Schematic representation of Dengue outbreak situation in Bhopal city during 2014-2016 and 2019- 2021.

Supplementary table S1. Patients’ clinical history at the time of OPD visit.

**Demographic and Clinical characteristics**	**SuspectedDFcasesregisteredfromwardno52-61 (n=300)**		
Age(years)	18-65		
Sex(Male/female)280	261/39		
Headache	50/300		
Myalgia	55/300		
Fever	280/300		
Daysoffeverinnumbers	5days=100	>5days=51	>10days=130
≤100degF	200		
≥100degF	80		
Arthralgia (%)	13.4		
Rash(%)	12		
Vomiting/Nausea(%)	6		
Retro-orbitalpain(%)	3		
Hematocrit(%)	20%		
Thromobocytopenia(%)[platelet count <=100000 cells/mm^2]	19-20%		

Supplementary Table S2: Mosquitoes pools infected with different serotypes.

**Mosquitoes Pools**	**Ward No.**	**Serotype**	**Co-infection detected in genomic sequencing**	**DENV infection**
Pool1	57	DENV2	DENV-1	DENV 2+1
Pool2	54	DENV2	DENV-1,3	DENV2+1,3
Pool3	59	DENV2	DENV-3	DENV 2+3
Pool4	58	DENV2	-	DENV2
Pool5	56	DENV2	DENV-1,3	DENV2+1,3
Pool6	52	DENV2	DENV-3	DENV 2+3
Pool7	55	DENV2	DENV-1,4	DENV2+1,4
Pool8	52	DENV2	DENV-1	DENV 2+1
Pool9	57	DENV2	DENV-3	DENV 2+3
Pool10	54	DENV2	DENV-1,3,4	DENV2+1,3,4
Pool11	61	DENV2	DENV-1	DENV 2+1
Pool12	58	DENV2	DENV-3	DENV 2+3
Pool13	55	DENV2	-	DENV2
Pool14	54	DENV2	-	DENV2
Pool15	57	DENV2	-	DENV2
Pool16	56	DENV2	DENV-1,3	DENV2+1,3
Pool17	60	DENV2	DENV-1	DENV 2+1
Pool18	57	DENV2	DENV-3	DENV 2+3

Supplementary Table S3: Serotype-specific viral load of DENV in host samples.

**Samples ID**	**Log_{10} RNA copies/ml**	**Serotype**
4	3.036949102	DENV-2
7	0.901185538	DENV-2
9	1.751891972	DENV-2
13	1.23861412	DENV-2
14	3.051788099	DENV-2
16	1.50918564	DENV-2
17	0.339549262	DENV-2
20	1.3269202	DENV-2
21	4.019290696	DENV-2
22	1.348271257	DENV-2
24	1.255592	DENV-2
27	0.01952341	DENV-2
30	1.84576456	DENV-2
32	2.594746995	DENV-3
34	2.980560914	DENV-2
36	2.24386111	DENV-1
38	5.206410447	DENV-2
39	1.85378795	DENV-1
41	1.52576455	DENV-2
42	3.087401692	DENV-2

45	1.7769875	DENV-2
46	1.754859771	DENV-2
47	2.23861107	DENV-2
50	2.155512687	DENV-2
51	6.078943463	DENV-2
58	1.229559282	DENV-2
61	3.2466127	DENV-2
67	7.758143463	DENV-2
71	1.909185339	DENV-2
72	6.592372756	DENV-1
73	4.868081318	DENV-2
76	4.250779047	DENV-2
78	2.43861166	DENV-4
79	2.692684375	DENV-2
85	2.214868675	DENV-2
92	4.182519662	DENV-2
93	2.487906218	DENV-2
94	3.348568037	DENV-2
97	3.176435673	DENV-2
99	3.244695059	DENV-2
101	3.057723698	DENV-2
102	1.80828016	DENV-2
103	1.85576495	DENV-3

105	5.087101392	DENV-2
110	5.797002523	DENV-2
115	5.6586131	DENV-2
117	2.867784538	DENV-2
121	2.099124499	DENV-2
134	3.23764109	DENV-2
149	4.23864532	DENV-2
168	3.33761105	DENV-2
185	5.23661187	DENV-2
210	2.32861135	DENV-2
213	1.3836564	DENV-2
220	1.5266882	DENV-2
223	1.79326711	DENV-2
225	2.03529017	DENV-2
226	1.053212657	DENV-2
229	1.07546923	DENV-2
232	1.21645282	DENV-2
235	0.8454518	DENV-2
240	0.22465407	DENV-1

241	0.4525266	DENV-2
247	1.6858375	DENV-3
250	1.2101275	DENV-2
251	1.7855439	DENV-2
257	0.5453656	DENV-2
260	1.0561318	DENV-2
261	1.2645047	DENV-2
270	0.4415456	DENV-2
271	0.546443	DENV-2
276	2.23662156	DENV-2
281	5.23861107	DENV-1
282	0.024552	DENV-2
284	1.7564823	DENV-2
289	0.6173541	DENV-3
290	1.5363548	DENV-2
293	0.5563366	DENV-2
296	1.5435275	DENV-2
299	0.264494	DENV-2
301	6.1386327	DENV-2
302	1.93161562	DENV-2
303	2.44861568	DENV-2
304	3.2036142	DENV-3
305	4.2561857	DENV-2
306	2.4658912	DENV-2
307	1.4526118	DENV-2

308	2.5916846	DENV-2
309	1.51692775	DENV-2
310	4.23891374	DENV-2
311	1.48668645	DENV-2
312	1.5642386	DENV-2
